# Instigation of endothelial Nlrp3 inflammasome by adipokine visfatin promotes inter‐endothelial junction disruption: role of HMGB1

**DOI:** 10.1111/jcmm.12657

**Published:** 2015-08-20

**Authors:** Yang Chen, Ashley L. Pitzer, Xiang Li, Pin‐Lan Li, Lei Wang, Yang Zhang

**Affiliations:** ^1^ Department of Pharmacology & ToxicologySchool of MedicineVirginia Commonwealth UniversityRichmondVAUSA; ^2^ Department of Pharmacological & Pharmaceutical SciencesCollege of PharmacyUniversity of HoustonHoustonTXUSA

**Keywords:** Nlrp3 inflammasome, visfatin, endothelium, tight junction proteins, obesity

## Abstract

Recent studies have indicated that the inflammasome plays a critical role in the pathogenesis of vascular diseases. However, the pathological relevance of this inflammasome activation, particularly in vascular cells, remains largely unknown. Here, we investigated the role of endothelial (Nucleotide‐binding Oligomerization Domain) NOD‐like receptor family pyrin domain containing three (Nlrp3) inflammasomes in modulating inter‐endothelial junction proteins, which are associated with endothelial barrier dysfunction, an early onset of obesity‐associated endothelial injury. Our findings demonstrate that the activation of Nlrp3 inflammasome by visfatin markedly decreased the expression of inter‐endothelial junction proteins including tight junction proteins ZO‐1, ZO‐2 and occludin, and adherens junction protein VE‐cadherin in cultured mouse vascular endothelial (VE) cell monolayers. Such visfatin‐induced down‐regulation of junction proteins in endothelial cells was attributed to high mobility group box protein 1 (HMGB1) release derived from endothelial inflammasome‐dependent caspase‐1 activity. Similarly, in the coronary arteries of wild‐type mice, high‐fat diet (HFD) treatment caused a down‐regulation of inter‐endothelial junction proteins ZO‐1, ZO‐2, occludin and VE‐cadherin, which was accompanied with enhanced inflammasome activation and HMGB1 expression in the endothelium as well as transmigration of CD43^+^ T cells into the coronary arterial wall. In contrast, all these HFD‐induced alterations in coronary arteries were prevented in mice with *Nlrp3* gene deletion. Taken together, these data strongly suggest that the activation of endothelial Nlrp3 inflammasomes as a result of the increased actions of injurious adipokines such as visfatin produces HMGB1, which act in paracrine or autocrine fashion to disrupt inter‐endothelial junctions and increase paracellular permeability of the endothelium contributing to the early onset of endothelial injury during metabolic disorders such as obesity or high‐fat/cholesterol diet.

## Introduction

Obesity is a major risk factor for cardiovascular diseases and has been strongly associated with endothelial dysfunction and coronary atherosclerosis. Obese patients have significantly elevated morbidity and mortality to coronary artery disease 1. Endothelial dysfunction is an early onset of obesity‐associated vascular diseases such as atherosclerosis, which was shown to be a trigger of vascular inflammation and consequent atherosclerotic lesions. However, it remains largely unknown how obesity instigates endothelial dysfunction. During obesity, adipose tissue as an active metabolic tissue secretes multiple metabolically important proteins known as ‘adipokines’. Some adipokines play a major role in insulin resistance and cardiovascular complications associated with obesity 2, 3. One of such novel adipokines is visfatin 4. Elevated plasma levels of visfatin were found in patients with type 2 and type 1 diabetes mellitus that can be lowered by regular physical exercise 5. Visfatin may be involved in the development of various obesity‐associated pathologies 6, 7, 8. It has been postulated that visfatin plays a role in inflammatory response during obesity. Visfatin expression and plasma levels of visfatin were also found to be associated with high‐fat diet (HFD)‐induced obesity in animal models 9. Therefore, it is of interest to use visfatin as a prototype adipokine to study obesity‐associated endothelial injury by exploring whether adipokines can directly cause endothelial cell dysfunction or injury.

NOD‐like receptor family pyrin domain containing 3 (Nlrp3) inflammasome is a major intracellular inflammatory machinery to switch on the inflammatory response to various danger signals or in diseases 10, 11, 12, 13, 14. Nlrp3 inflammasome consists of a proteolytic complex formed by three main components including Nlrp3 that functions as a pattern recognition receptor, the adaptor protein (Adapter Protein Apoptosis‐associated Speck‐like Protein) ASC and caspase‐1 15, 16. When the Nlrp3 inflammasome complex is formed, caspase‐1 is activated to cleave its substrates including the precursors of inflammatory cytokine interleukin (IL)‐1β 17, 18. Nlrp3 detects endogenous stress‐associated danger signals such as ATP and β‐amyloid to produce local tissue sterile inflammation 10, 11. In this respect, the inflammasome has been implicated in the pathogenesis of various metabolic diseases including diabetes mellitus 15, 19, 20. Recently, we demonstrated that the injurious adipokine visfatin activates the Nlrp3 inflammasome in cultured endothelial cells *via* up‐regulation of reactive oxygen species (ROS) 21. However, the pathological relevance of endothelial Nlrp3 inflammasome activation in obesity‐associated vasculopathy remains unclear.

Endothelium is the monolayer of endothelial cells that lines the interior surface of blood and lymphatic vessels. Endothelium normally serves as a permeability barrier to the movement of fluid and proteins from the intravascular compartment to the interstitium. Endothelium barrier dysfunction results in many pathological consequences including endothelial cell injury, increased leakage of plasma proteins to the interstitial compartment and enhanced leucocyte transmigration in the vasculature 22. In the endothelium, endothelial cells are connected to each other by a complex set of junctional proteins including tight junction and adherens junction proteins 23, 24, 25. The typical tight junction proteins in endothelial cells include ZO‐1/2 and occludin 26, 27, and the typical adherens junction proteins is vascular endothelial (VE) cadherin. These inter‐endothelial junction proteins play critical roles in modulating paracellular permeability of inter‐endothelial junctions 22, 28, 29. In this regard, disruption of inter‐endothelial junctions between endothelial cells occurs in the early stages of endothelial barrier dysfunction. In this study, we demonstrate for the first time a novel role of endothelial Nlrp3 inflammasome in visfatin‐ and HFD‐induced inter‐endothelial junction disruption. Our findings show that the activation of endothelial Nlrp3 inflammasome by visfatin in cultured endothelial cells or by HFD in coronary arterial endothelium decreases the expression of inter‐endothelial junction proteins including ZO‐1, ZO‐2, occludin and VE‐Cadherin. Such an action of endothelial Nlrp3 inflammasome is mostly attributed to the increased release of high mobility group box 1 (HMGB1), one of the major damage‐associated molecular patterns (DAMPs) 30. In addition, HFD‐induced endothelial junction disruption is associated with enhanced T cell transmigration in mouse coronary arterial walls, which was attenuated in mice with Nlrp3 gene deletion. Our study implicates the clinical potential of targeting inflammasome and HMGB1 signalling axis for the prevention of the early onset of obesity‐associated vasculopathy.

## Materials and methods

### Animal procedures

Eight‐week‐old male C57BL/6J wild‐type (Nlrp3^+/+^) and Nlrp3^−/−^ mice were used in this study (The Jackson Laboratory, Bar Harbor, Maine, USA). Nlrp3 knockout (*Nlrp3* ^−/−^) and wild‐type (*Nlrp3* ^+/+^) mice were genotyped following the protocol by the vendor. For 6 weeks, mice were fed either a normal diet (ND) or a HFD (60 kcal % fat; Research Diets, New Brunswick, NJ, USA) as we reported previously 31. All protocols were approved by the Institutional Animal Care and Use Committee of Virginia Commonwealth University. After 6 weeks, mice were killed and heart tissues were harvested for immunofluorescence or biochemical examinations.

### Cell culture

The mouse vascular endothelial cell (MVEC) line EOMA was purchased from ATCC and cultured as we recently described 21, 32. Mouse vascular endothelial cells were cultured in DMEM (Gibco, Grand Island, NY, USA), containing 10% of foetal bovine serum (Gibco) and 1% penicillin–streptomycin (Gibco). The cells were cultured in a humidified incubator at mixture at 37°C with 5% CO_2_ and 95% air. Cells were passaged by trypsinization [Trypsin/ethylenediaminetetraacetic acid (EDTA); Sigma‐Aldrich, St. Louis, MO, USA], followed by dilution in DMEM medium containing 10% foetal bovine serum.

### Confocal microscopic analysis of MVECs

For colocalization in MVECs, cells were grown on glass coverslips and then treated with 4 μg/ml visfatin (BioVision, Mountain View, CA, USA) for 24 hrs and was then terminated by the fixation of the cells in 4% (Paraformaldehyde) PFA for 15 min. as previously described 33. Cells were washed in PBS and cells were incubated for 2 hrs at 4°C with rabbit anti‐ZO‐1 (1:500; Invitrogen, Grand Island, NY, USA), anti‐ZO‐2 (1:500; Invitrogen), anti‐Occludin (1:1000; Abcam, Cambridge, MA, USA), anti‐VE‐Cadherin (1:1000; Abcam). Double immunofluorescent staining was performed by Alexa Fluor 488 or Alexa Fluor 555‐labelled secondary antibody (1:100; Invitrogen) incubation for 1 hr at room temperature. The slices were visualized through sequentially scanning on an Olympus laser scanning confocal microscope (Fluoview FV1000; Olympus, Center Valley, PA, USA).

### Immunoblotting

Mouse vascular endothelial cells were treated as indicated before incubation with visfatin. Microsomes were isolated as previously described with modifications 34. Cells were washed twice with PBS and scraped in lysis buffer containing a protease inhibitor cocktail (Roche, Nutley, NJ, USA), 255 mM sucrose, 1 mM (Phenylmethanesulfonyl flouride) PMSF, 1 mM Na_3_VO_4_ and 1 mM EDTA. The nuclei and cell debris of the lysates were spun down (5000 × g for 5 min. at 4°C) and the supernatant (termed homogenate) containing the microsome and cytosolic fractions were collected. Microsomes and cytosols were separated by a differential centrifugation of the homogenate at 10,000 × g for 20 min. and then at 100,000 × g for 90 min. The pellet (microsomes) was resuspended in lysis buffer and the supernatant (cytosol) was also collected. The samples were denatured with reducing Laemmli SDS‐sample buffer and boiled for 5 min. Samples were run on SDS‐PAGE gel, transferred into (Polyvinylidene Flouride) PVDF membrane and blocked. The membranes were probed with primary antibody of anti‐HMGB1 (1:1000; Abcam) or anti‐ZO‐1 (1:1000; Invitrogen), anti‐ZO‐2 (1:1000; Invitrogen), anti‐Occludin (1:1000; Abcam), anti‐VE‐Cadherin (1:1000; Abcam) overnight at 4°C followed by incubation with secondary antibody and then conjugated to horseradish peroxidase‐labelled immunoglobulin G. The immunoreactive bands were enhanced by chemiluminescence methods and imaged on Kodak Omat (Rochester, New York, USA) film. β‐actin served as a loading control. The intensity of the bands was quantified by densitometry using ImageJ 6.0 (NIH, Bethesda, MD, USA).

### Nucleofection

Transfection of shRNA plasmids was performed with a 4D Nucleofector X‐Unit (Lonza, Allendale, NJ, USA) according to the manufacturer's instructions as previously described 35. The plasmid encoding shRNA for mouse Nlrp3 gene was obtained from Origene (#TG510752; Rockville, MD, USA). Briefly, MVECs were tyrpsinized and centrifuged at 80 × g for 10 min. The cell pelletet was resuspended in 100 μl SF Nucleofection solution (Lonza) for Nucleofection (with the program code DS198). The program was chosen based on the fact that over 80% of Nucleofected cells were positive for (Green Fluorescent Protein) GFP control plasmid as analysed by flow cytometry (GUAVA, Hayward, CA, USA). For each Nucleofection sample (10^6^ cells/sample), 2 μg plasmid DNA was added in 100 μl SF Nucleofection solution. After Nucleofection, cells were cultured in DMEM medium for 24 hrs. Neucleofection of Nlrp3 shRNA plasmids resulted in a decrease in the Nlrp3 protein expression as examined by immunoblotting analysis using antibody against Nlrp3 (Abcam; data not shown).

### RNA interference

Small interference RNAs (siRNAs) for advanced glycation end products (RAGE) and toll‐like receptor 4 (TLR4) were commercially available (Santa Cruz Biotechnology, Santa Cruz, CA, USA). The sequence for RAGE siRNA and TLR4 siRNA were confirmed to be effective in silencing RAGE gene or TLR4 gene in different cells by the company. The scrambled small RNA has been also confirmed as non‐silencing double stranded RNA and was used as a control in this study. Transfection of siRNA was performed with the siLentFect Lipid Reagent (Bio‐Rad, Hercules, CA, USA) according to the manufacturer's instructions. The effects of RNA interference on the expression of the targeted proteins were examined by immunoblotting analysis using antibody against RAGE or TLR4 (Fig. S1).

### Confocal analysis of protein expression in coronary arterial endothelium

Confocal immunofluorescence analysis was performed to detect colocalization of different proteins in the endothelium of coronary arteries as we previously described 36. Briefly, the mouse hearts were frozen in Tissue‐Tek OCT and cut by cryostat into 10 μm sections and mounted on Superfrost/Plus slides. After fixation with acetone, the frozen section slides were incubated with following primary antibodies: rabbit anti‐ZO‐1 (1:100; Invitrogen), anti‐ZO‐2 (1:100; Invitrogen), anti‐Occludin (1:100; Abcam), anti‐VE‐Cadherin (1:100; Abcam), or anti‐HMGB1 (1:500; Abcam), or anti‐CD43 (1:100; BD, East Rutherford, NJ, USA). Endothelium was also visualized by costaining slides with sheep anti‐von Willebrand factor (vWF) against endothelial cell marker vWF (1:200; Abcam). After incubation with primary antibodies, the slides were washed and labelled with corresponding Alexa Fluor‐488 and Alexa Fluor‐555 conjugated secondary antibodies (Invitrogen). Then, the slides were washed, mounted and visualized through sequentially scanning on an Olympus laser scanning confocal microscope (Fluoview FV1000; Olympus). Colocalization was analysed by Image Pro Plus software, and the colocalization coefficient was represented by Pearson's correlation coefficient as previously reported 37.

### *In situ* analysis of caspase‐1 activity in coronary arterial endothelium

This assay uses Fluorescent Labeled Inhibitor of Caspases (FLICA^™^) probes (ImmunoChemistry Technologies, LLC, Bloomington, MN, USA) to label active caspase‐1 enzyme in coronary arterial endothelium 32, 38. The FLICA probes are comprised of a caspase‐1 recognition sequence tyrosine‐valine‐alanine‐aspartic acid (YVAD) that binds to active caspase‐1, a fluoromethyl ketone (FMK) moiety that results in irreversible binding with the enzyme, and a fluorescent tag FAM (carboxyfluorescein) reporter. After entering the cells, the FLICA reagent FAM‐YVAD‐FMK becomes covalently coupled to the active caspase‐1, while any unbound FLICA reagent diffuses out of the cell and is washed away. The remaining green fluorescent signal is a direct measure of the active caspase‐1 enzyme activity in the cell or tissue samples. To detect caspase‐1 activity in coronary arterial endothelium, frozen section slides were first fixed in acetone then incubated overnight at 4°C with sheep anti‐vWF (1:200; Abcam). Slides were then costained with fluorescence‐conjugated anti‐sheep secondary antibody and FLICA reagent (1:10) from a FLICA^™^ Caspase 1 Assay Kit (ImmunoChemistry Technologies, LLC) for 1.5 hrs at room temperature. The slides were washed, mounted, visualized and analysed by confocal microscopy as described above. Colocalization was analysed by Image Pro Plus software, and the colocalization coefficient was represented by Pearson's correlation coefficient.

### Statistics

Data are presented as means ± SE. Significant differences between and within multiple groups were examined using anova for repeated measures, followed by Duncan's multiple‐range test. A Students’ *t*‐test was used to detect significant difference between two groups. The statistical analysis was performed by SigmaStat 3.5 software (Systat Software, San Jose, CA, USA). *P* < 0.05 was considered statistically significant.

## Result

### Visfatin‐induced disruption of tight and adherens junction proteins in MVECs is dependent on Nlrp3 inflammasome activation

**Figure 1:**
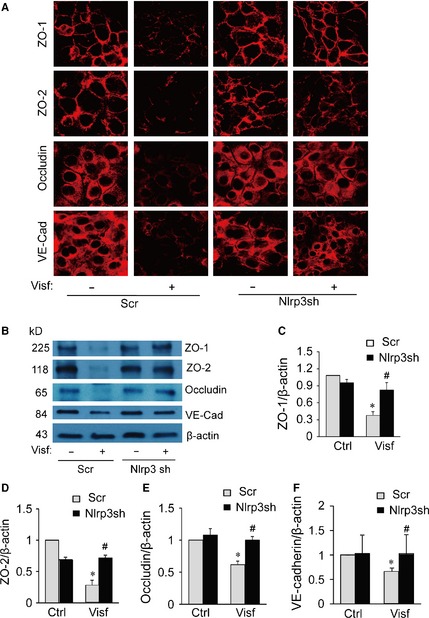
Effects of Nlrp3 gene silencing on visfatin‐induced disruption of junction proteins in mouse vascular endothelial cells (MVECs). MVECs were transfected scramble (Scr) or Nlrp3 shRNA (Nlrp3sh) plasmids by Nucleofection and then stimulated with or without visfatin (Visf: 4 μg/ml) for 24 hrs. (**A**) Immunofluorescence stainings were performed with Alexa555‐conjugated antibodies against ZO‐1, ZO‐2, occludin or VE‐Cadherin (VE‐Cad) for determination of the expression of these junction proteins. Representative images show the cell membrane of fluorescence of ZO‐1, ZO‐2, occludin or VE‐Cadherin (red) are representative of at least three independent experiments. (**B**–**F**) Representative Western blot gel document and summarized data showing the protein expression of ZO‐1, ZO‐2, occludin, VE‐Cadherin and β‐actin expression in the microsomes of MVECs (*n* = 3–4). *P < 0.05 *versus* Scr; ^#^ *P* < 0.05 *versus* Scr+Visf.

Our recent studies have demonstrated that injurious adipokine visfatin activates Nlrp3 inflammasome in endothelial cells. However, such a role of visfatin‐induced Nlrp3 inflammasome activation in regulation of inter‐endothelial junction proteins remains unknown. Here, we first determined whether Nlrp3 inflammasome activation by visfatin causes disassembly of junction proteins in cultured MVECs *in vitro*. We demonstrated that in the treatment of confluent MVECs with visfatin markedly decreased the expression of tight junction proteins ZO‐1, ZO‐2 and Occludin and adherens junction protein VE‐Cadherin at cell junctions in endothelial cell monolayers (Fig. 1A). Such visfatin‐induced decreases in tight and adherens junction proteins were further confirmed by quantitative immunoblotting analyses (Fig. 1B). However, the silencing of the *Nlrp3* gene by transfecting MVECs with the Nlrp3 shRNA plasmids prevented visfatin‐induced decreases in protein expression of ZO‐1, ZO‐2, Occludin and VE‐Cadherin (Fig. 1A and B). Thus, these results indicate that the activation of Nlrp3 inflammasome by visfatin causes disruption of junction proteins in endothelial cell monolayers.

### Role of HMGB1 in visfatin‐induced tight and adherens junction protein disruption in MVECs

**Figure 2:**
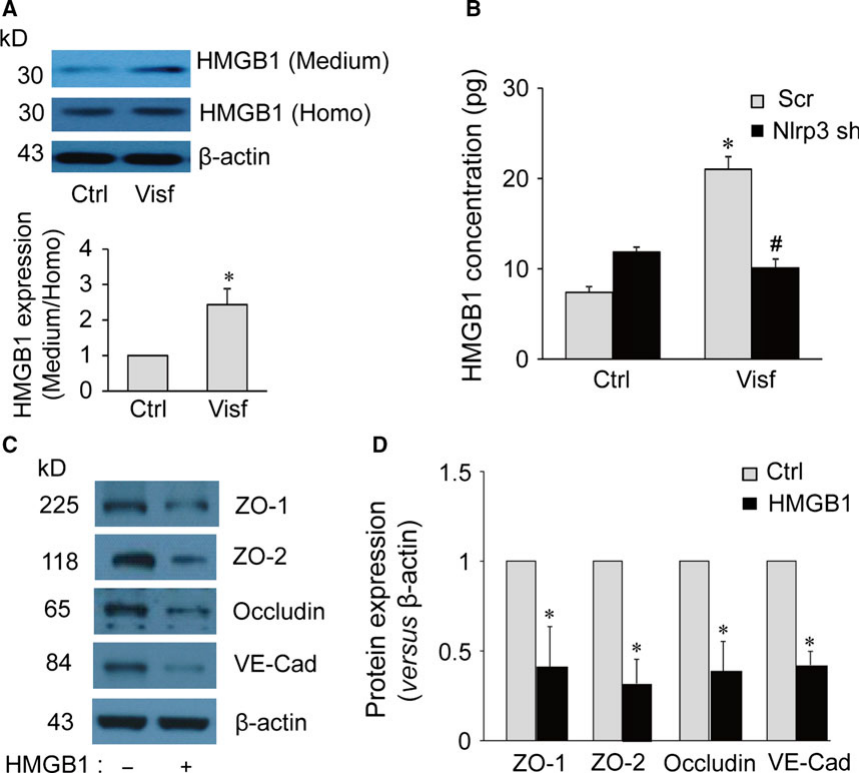
(**A**) Western blot documents and summarized data showing the effect of control or visfatin (0 or 4 μg/ml for 24 hrs) on the expression of high mobility group box protein 1 (HMGB1) or β‐actin in either cell culture medium (Medium) or homogenized cytoplasm (Homo) of mouse vascular endothelial cells (MVECs; *n* = 6). *P < 0.05 *versus* control. (**B**) MVECs transfected with scramble shRNA (Scr) or Nlrp3 shRNA (Nlrp3sh) plasmids and then stimulated with or without visfatin (Visf: 4 μg/ml) for 24 hrs. Summarized data show the concentration of HMGB1in culture medium of MVECs as analysed by ELISA (*n* = 6). *P < 0.05 *versus* Scr; ^#^ *P* < 0.05 *versus* Scr+Visf. (**C**) Cells were incubated with fresh culture medium before addition of recombinant HMGB1 (15 μg/ml) for 24 hrs. Representative Western blot gel document and summarized data show the effect of recombinant HMGB1 (*n* = 3) on protein expression of ZO‐1, ZO‐2, occludin, VE‐Cadherin and β‐actin in the microsomes of MVECs. *P < 0.05 *versus* control.

HMGB1 is an evolutionarily conserved non‐histone chromatin‐binding protein and is a nuclear protein physiologically involved in the maintaining of DNA structure in the nucleus. High mobility group box 1 is one of the major DAMPs 30, which can be derived from Nlrp3 inflammasome activation 39. Recent studies have demonstrated that HMGB1 alone increases permeability of the endothelial cell monolayers 40. Therefore, we test the hypothesis that upon visfatin stimulation, HMGB1 is released from MVECs as an autocrine or paracrine to cause junction protein disruption in endothelial cell monolayers. As shown in Figure 2A, visfatin treatment increased HMGB1 release from MVECs as shown by the increased protein expression of HMGB1 in the culture medium. Quantification of HMGB1 release by ELISA (MyBioSource, San Diego, CA, USA) further demonstrated that Nlrp3 gene silencing abolished the visfatin‐induced HMGB1 release from MVECs (Fig. 2B). We also noticed that the HMGB1 could be detected in control group, which suggests a basal level of the HMGB1 release in endothelial cells under control condition. Our finding is in consistence with a recent study showing that the basal level of HMGB1 also could be found by WB in normal condition medium of the endothelial cell cultures 41. These results suggest that visfatin‐induced HMGB1 release requires Nlrp3 inflammasome functionality. Indeed, treatment of MVECs with recombinant HMGB1 (purchased from Abcam) decreased the protein expression of ZO‐1, ZO‐2, occludin and VE‐Cadherin in these cells (Fig. 2C and D). As a comparison, recombinant IL‐1β did not decrease the expression of these junction proteins (data not shown). Yang *et al*. has reported HMGB1 might be a key target of redox modifications during inflammation, and activities of HMGB1 require both reduced C106 and the formation of an intramolecular disulphide bond between C23 and C45 42. Visfatin increases the production of ROS in endothelial cells 43. Therefore, it is possible that HMGB1 released by the endothelial cells is in a similar redox regulated form as reported by Yang *et al*.

**Figure 3:**
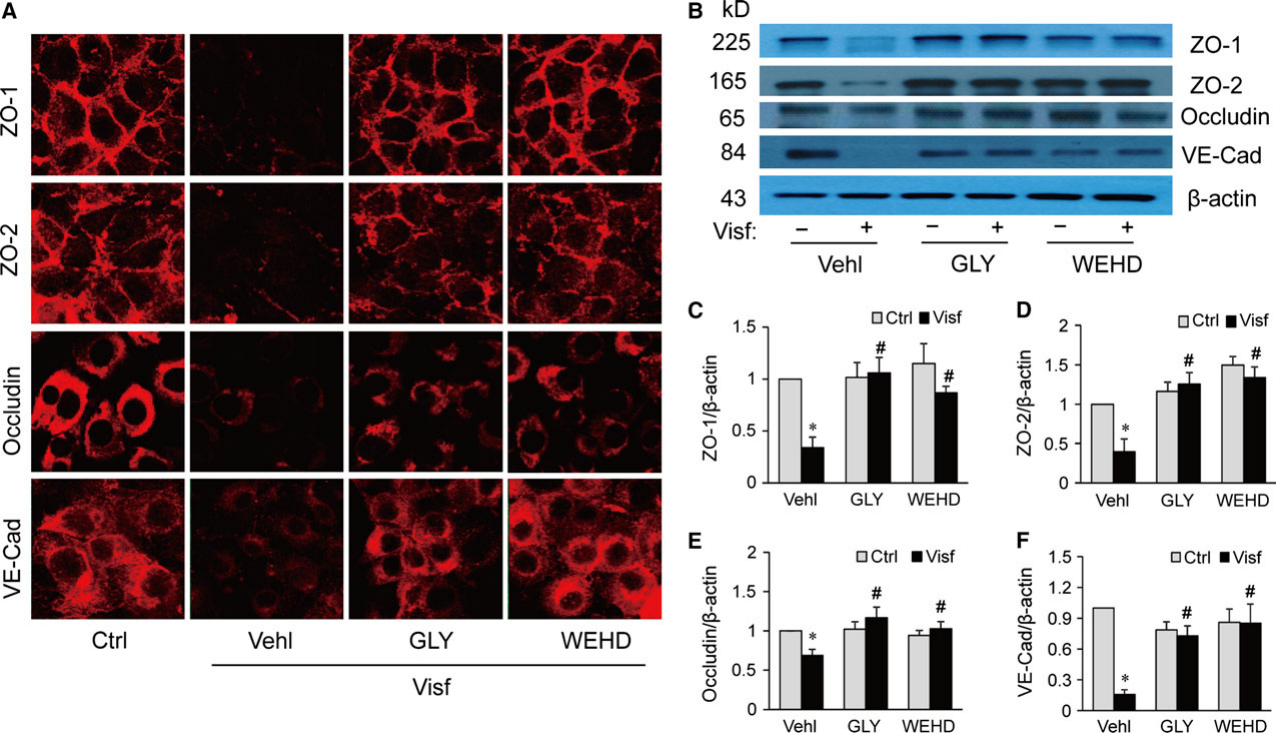
Inhibition of high mobility group box protein 1 (HMGB1) or caspase‐1 activity prevents visfatin‐induced disruption of junction proteins in mouse vascular endothelial cells (MVECs). MVECs were stimulated with or without visfatin (Visf, 4 μg/ml) for 24 hrs in the presence of PBS (Vehl: vehicle), HMGB1 inhibitor glycyrrhizin (GLY, 130 μmol/l) or caspase‐1 inhibitor Z‐WEHD‐fluoromethyl ketone (FMK) (WEHD, 0.2 μg/ml). (**A**) Immunofluorescence stainings were performed with Alexa555‐conjugated antibodies against ZO‐1, ZO‐2, occludin or VE‐Cadherin (VE‐Cad) for determination of the expression of these junction proteins. Representative images show the cell membrane of fluorescence of ZO‐1, ZO‐2, occludin or VE‐Cadherin (red) are representative of at least three independent experiments. (**B**–**F**) Representative Western blot gel document and summarized data showing the protein expression of ZO‐1, ZO‐2, occludin, VE‐Cadherin and β‐actin expression in the microsomes of MVECs (*n* = 4–5). *P < 0.05 *versus* Vehl Ctrl; ^#^ *P* < 0.05 *versus* Visf alone.

To further confirm the role of endothelial cell‐derived HMGB1 in visfatin‐induced disassembly of junction proteins, confluent endothelial cell monolayers were treated with visfatin in the presence of HMGB1 activity inhibitor glycyrrhizin or caspase‐1 inhibitor WEHD. It was found that glycyrrhizin and WEHD prevented visfatin‐induced disruption of ZO‐1, ZO‐2, Occludin and VE‐Cadherin at cell junctions in the endothelial cell monolayer (Fig. 3A) and decreases in their total protein expression (Fig. 3B). These results suggest that visfatin‐induced disruption of junction proteins is dependent on caspase‐1 activation in and HMGB1 release by endothelial cells.

### RAGE but not TLR4 mediates visfatin‐induced down‐regulation of tight and adherens junction proteins in MVECs

**Figure 4:**
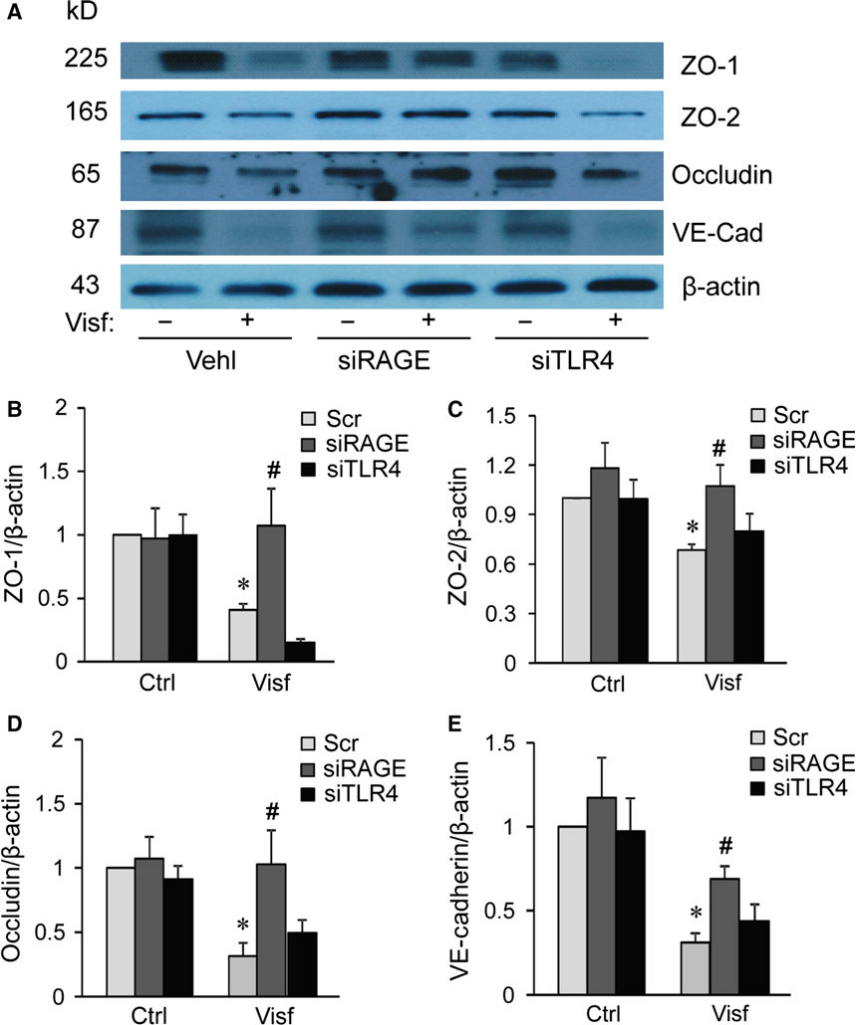
High mobility group box protein 1 (HMGB1)‐induced endothelial junction disruption effect is mediated by the RAGE. Mouse vascular endothelial cells (MVECs) were transfected with scramble (Scr), RAGE siRNA (siRAGE) or TLR4 siRNA (siTLR4) for 4 hrs and then stimulated with or without Visfatin (4 μg/ml, 24 hrs). (**A**‐**E**) Representative Western blot gel document and summarized data showing the protein expression of ZO‐1, ZO‐2, occludin, VE‐Cadherin and β‐actin expression in the microsomes of MVECs (*n* = 4–5). *P < 0.05 *versus* Scr Ctrl; ^#^ *P* < 0.05 *versus* Scr+Visf.

Previous studies showed that pro‐inflammatory activity of HMGB1 is primarily mediated *via* two transmembrane receptors: receptor for RAGE or TLR4 40, 44, 45, 46. Thus, we examined whether the silencing of the HMGB1 receptor (s) in endothelial cells could abolish the visfatin‐induced disruption of tight and adherens junction proteins. We found that silencing RAGE, but not TLR4 significantly reversed visfatin‐induced down‐regulation of ZO‐1, ZO‐2, occludin and VE‐cadherin in the endothelial cell monolayers (Fig. 4). Similarly, RAGE silencing prevents down‐regulation of inter‐endothelial junction proteins induced by recombinant HMGB1 (Fig. S2). Together, these data suggest that visfatin‐induced disruption on junction proteins is primarily through HMGB1‐RAGE signalling pathway.

### Nlrp3 deficiency abolishes HFD‐induced disassembly of tight junction and adherens junction proteins in coronary arterial endothelium

**Figure 5:**
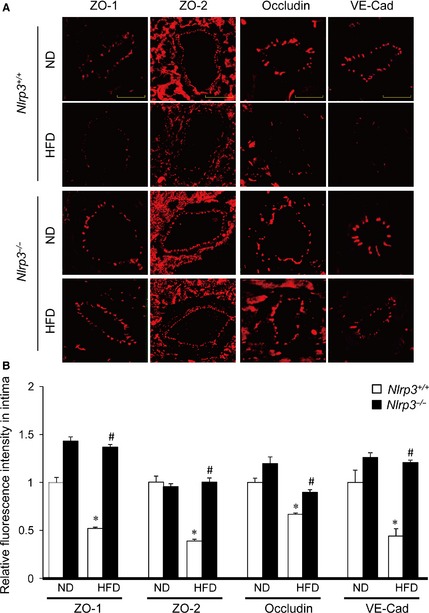
Nlrp3 gene deletion inhibits high‐fat diet (HFD)‐induced disassembly of tight junction and adherens junction proteins in mouse coronary arterial endothelium. Wild‐type (*Nlrp3* ^+/+^) or Nlrp3 knockout (*Nlrp3* ^−/−^) Mice were fed with either a normal diet (ND), or a high‐fat diet (HFD) for 6 weeks. (**A**) Frozen sections of mouse hearts were stained with Alexa555‐conjugated antibodies against ZO‐1, ZO‐2, Occludin, or VE‐Cadherin; scale bar = 50 μm. (**B**) The summarized data show the fluorescence intensity of the endothelial layer (*n* = 4–6). *P < 0.05 *versus* ND on *Nlrp3* ^+/+^; ^#^ *P* < 0.05 *versus Nlrp3* ^+/+^ with HFD.

Next, we examined the role of Nlrp3 inflammasome in the disassembly of endothelial junction proteins in the coronary endothelium in a HFD‐induced obesity model using *Nlrp3* ^+/+^ and *Nlrp3* ^−/−^ mice. As shown in Figure 5A, immunofluorescence studies demonstrated that the treatment of *Nlrp3* ^+/+^ mice with HFD markedly decreased the expression of tight junction protein ZO‐1, ZO‐2, and Occludin and adherens junction protein VE‐Cadherin in the endothelium layer of coronary arteries. In contrast, such HFD‐induced down‐regulation of junction proteins was significantly reversed in *Nlrp3* ^−/−^ mice.

### Nlrp3 deficiency inhibits HFD‐induced inflammasome activation and HMGB1 expression in coronary arterial endothelium

**Figure 6:**
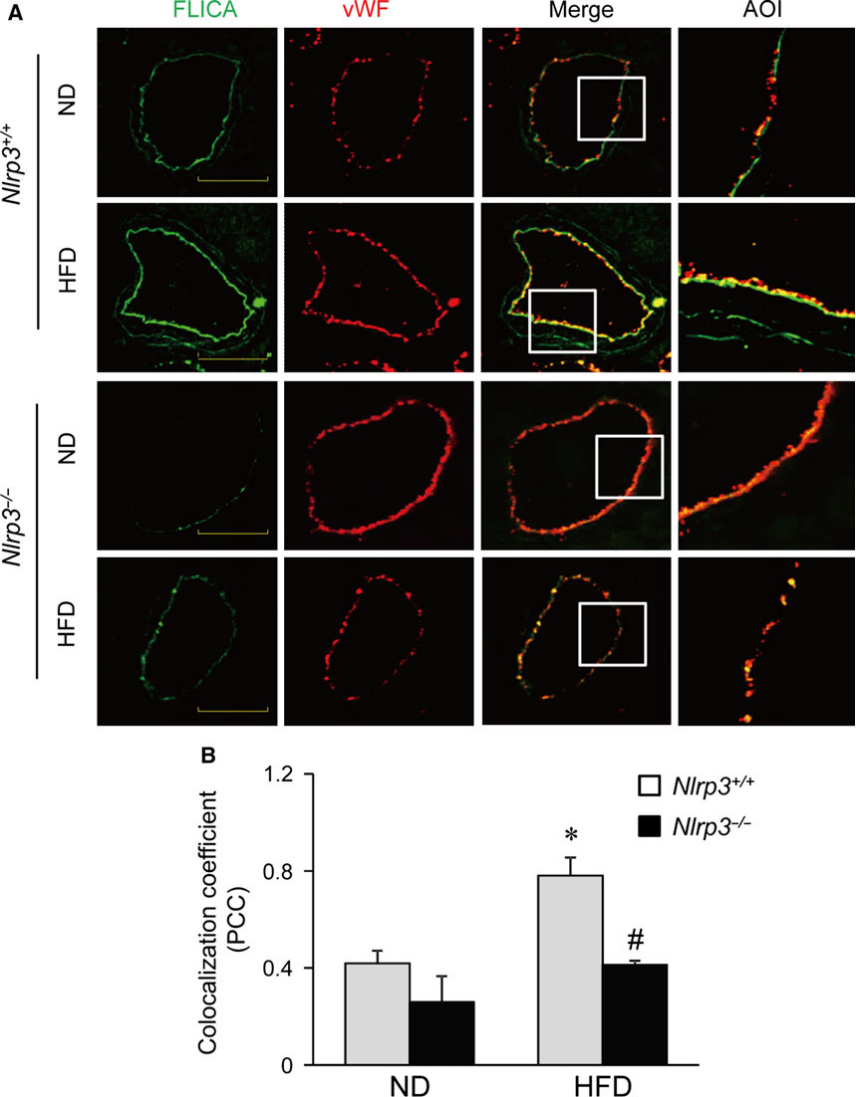
Nlrp3 deficiency gene deletion inhibits high‐fat diet (HFD)‐induced inflammasome activation in mouse coronary arterial endothelium. Wild‐type (Nlrp3^+/+^) or Nlrp3 knockout (Nlrp3^−/−^) Mice were fed with normal diet (ND) and high‐fat diet (HFD) for 6 weeks. Frozen sections of mouse hearts were stained with FLICA, a green fluorescent probe specific for active caspase‐1, and Alexa555‐conjugated antibodies against an endothelium marker vWF in coronary arteries. (**A**) The merged images displayed yellow dots or patches indicating the colocalization of FLICA (green) with vWF (red). Enlarged images of area of interest (AOI) in merged images are shown; scale bar = 50 μm. (**B**) The summarized data show the colocalization coefficient of FLICA with vWF (*n* = 5–7). *P < 0.05 *versus Nlrp3* ^+/+^ with ND; ^#^ *P* < 0.05 *versus Nlrp3* ^+/+^ with HFD.

We further examined whether the HFD‐induced disassembly of the endothelial junctions was accompanied by the activation of the endothelial Nlrp3 inflammasome and HMGB1 in the coronary arteries of *Nlrp3* ^+/+^ or *Nlrp3* ^−/−^ mice. To this end, the endothelial inflammasome activation was first analysed by staining the coronary arteries with FLICA, a green fluorescent probe that specifically bind to the active form of caspase‐1. The endothelium was visualized by staining the coronary arteries with an endothelial cell marker vWF. As shown in Figure 6A and B, immunofluorescence studies demonstrated that the treatment of mice with HFD markedly increased capase‐1 activity in coronary arteries of *Nlrp3* ^+/+^ mice as shown by the enhanced green fluorescence of FLICA. Further, in these arteries, HFD increased the colocalization of green FLICA fluorescence with vWF suggesting that HFD increased caspase‐1 activity primarily in the arterial endothelium. In contrast, such HFD‐induced activation of endothelial caspase‐1 was abolished in the coronary arteries of *Nlrp3* ^−/−^ mice. These results suggest that Nlrp3 inflammasome is activated in the coronary arterial endothelium in mice with HFD.

**Figure 7:**
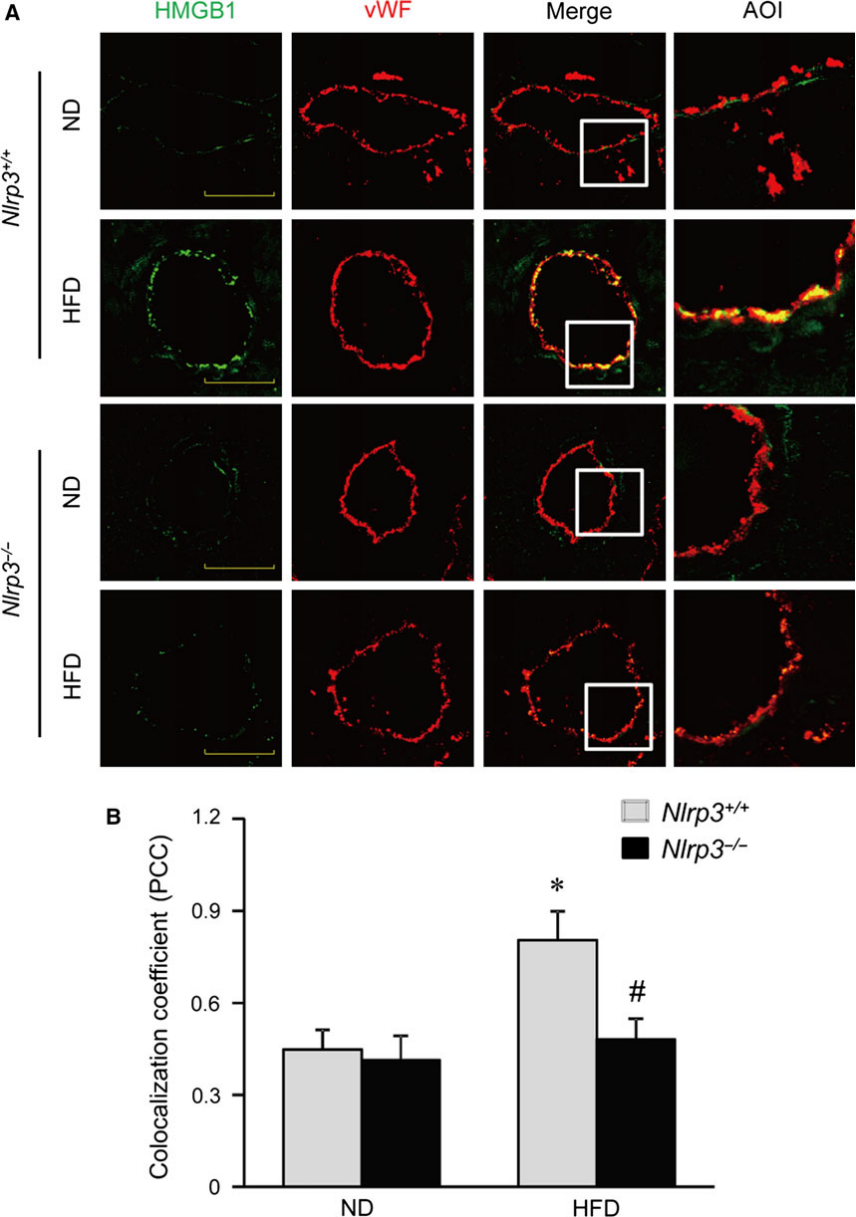
Nlrp3 deficiency inhibits high‐fat diet (HFD)‐induced increases of high mobility group box protein 1 (HMGB1) release in coronary arterial endothelium. Wild‐type (Nlrp3^+/+^) or Nlrp3 knockout (Nlrp3^−/−^) Mice were fed with normal diet (ND) and high‐fat diet (HFD) for 6 weeks. Frozen sections of mouse hearts were used for confocal immunofluorescent analysis. (**A**) Representative confocal fluorescence images of HMGB1 with endothelium marker vWF in coronary arteries of mice. Enlarged images of area of interest (AOI) in merged images are shown; scale bar = 50 μm. (**B**) The summarized data show the colocalization coefficient (PCC) of HMGB1 with vWF (*n* = 4–8). *P < 0.05 *versus Nlrp3* ^+/+^ with ND; ^#^ *P* < 0.05 *versus Nlrp3* ^+/+^ with HFD.

Given the role of HMGB1 in visfatin‐induced endothelial junction disruption, we further examined whether HMGB1 expression is also up‐regulated in the coronary arterial endothelium of mice treated with HFD. As shown in Figure 7A and B, HFD treatment increased the expression of HMGB1 in coronary arterial endothelium (vWF) of *Nlrp3* ^+/+^ mice as shown by increased yellow staining and colocalization coefficient between vWF and HMGB1. However, such HFD‐induced increases in the colocalization of HMGB1 with vWF were suppressed in the endothelium layer in coronary arteries of *Nlrp3* ^−/−^ mice.

### Nlrp3 deficiency prevents HFD‐induced CD43^+^ T cell adhesion and infiltration in coronary arteries

**Figure 8:**
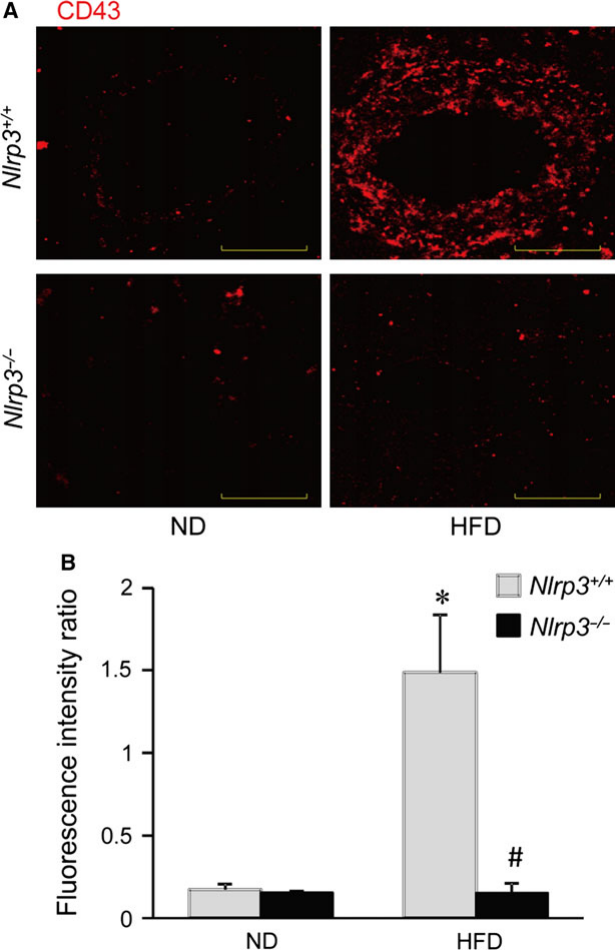
Nlrp3 deficiency blocks high‐fat diet (HFD)‐induced T cell adhesion and infiltration in coronary arterial wall. Wild‐type (*Nlrp3* ^+/+^) or Nlrp3 knockout (*Nlrp3* ^−/−^) Mice were fed with either a normal diet (ND), or a high‐fat diet (HFD) for 6 weeks. (**A**) Frozen sections of mouse hearts were stained with T cell marker (CD43). The fluorescence images displayed red dots indicating the CD43‐positive cells infiltrated into the arterial wall. (**B**) Summarized data show the fluorescence intensity of the arterial wall area (*n* = 4). *P < 0.05 *versus Nlrp3* ^+/+^ with ND; ^#^ *P* < 0.05 *versus Nlrp3* ^+/+^ with HFD.

We next determined whether the disruption of tight and adherens junction was associated with enhanced T cell adhesion and infiltration in coronary arteries by staining coronary arteries with CD43^+^ antibodies. CD43 is expressed on all T cells and can be used as a pan T cell maker. As shown in Figure 8, HFD treatment resulted in massive increases in red fluorescence staining for CD43 in coronary arteries of *Nlrp3* ^+/+^ mice. It should be noted that such staining for CD43 was in a punctated pattern indicating the presence of CD43^+^ T cells in and around the arterial walls of coronary arteries. However, such HFD‐induced increases in CD43^+^ T cells were abolished in the coronary arteries of *Nlrp3* ^−/−^ mice. Thus, these data suggest that HFD treatment induces T cell adhesion and infiltration in coronary arteries, which can be prevented by Nlrp3 gene deletion.

## Discussion

Our recent studies have demonstrated that injurious adipokine visfatin activates Nlrp3 inflammasome in endothelial cells *via* ROS‐dependent mechanism 21. However, the pathological relevance of such Nlrp3 inflammasome activation in modulating endothelial cell functions remains largely unknown. This study is aimed to determine whether visfatin‐induced Nlrp3 inflammasome activation results in endothelial dysfunction or injury by disrupting inter‐endothelial junctions. Our data reveal that activation of Nlrp3 inflammasome by either visfatin or HFD results in the down‐regulation of tight and adherens junction proteins in cultured endothelial monolayers or coronary arterial endothelium, which is associated with Nlrp3‐dependent HMGB1 production and its receptor RAGE‐mediated signalling in the endothelial cells.

Endothelium forms an interface and serves as a barrier between circulating blood or in the lumen and the rest of the vessel wall. Under the physiological condition, the endothelium functions as a selective barrier through inter‐endothelial junctions, which allows the convective and diffusive transport of molecules of less than 3 nm in diameter 22, 47. The permeability of inter‐endothelial junctions is determined by the adhesive properties of tight junction proteins and adherens junction proteins 28, 29, 48. Decreased expression of tight junction proteins ZO‐1/2 and occludin reduces the adhesive properties of these proteins, which has been considered as a marker event for enhanced permeability of inter‐endothelial junction during vascular dysfunction. In addition to tight junction proteins, the adherens junction protein VE‐Cadherin also play a crucial role in modulating inter‐endothelial junctional permeability 49. The present study demonstrated that stimulation of endothelial cells with visfatin decreased the expressions of tight junction protein ZO‐1/2, occludin, and the adherens junction protein VE‐Cadherin in cultured endothelial monolayers, whereas *Nlrp3* gene silencing prevented such visfatin‐induced down‐regulation of junction proteins. These data, for the first time, reveal a critical role of endothelial Nlrp3 inflammasome in modulating inter‐endothelial junction integrity 50.

Our data further confirmed that visfatin‐induced inter‐endothelial junction disruption is primarily associated with HMGB1 release by endothelial cells. High mobility group box 1, a ubiquitous nuclear and cytosolic protein, is released into the extracellular space by immune cells during sterile inflammation and infection, and passively released by damaged or necrotic cells 30, 39. In the present study, we observed that visfatin induced HMGB1 release from the endothelial cells, which was abolished by Nlrp3 gene silencing. The reduced capacity of endothelial cells transfected with Nlrp3 shRNA to release HMGB1 is consistent with recent findings that Nlrp3 inflammasome activation could lead to translocation and secretion of HMGB1 by immune cells 39. Further, direct treatment of endothelial cell monolayers with recombinant HMGB1 but not IL‐1β decreased tight and adherens junction protein expression. Similarly, a recent study also demonstrated that HMGB1 alone decreased the expression of membrane‐associated VE‐Cadherin in and increased the permeability of the monolayers of the human umbilical vein endothelial cells 40. Moreover, HMGB1 increases the permeability of caco‐2 enterocytic monolayers and impairs intestinal barrier function in mice 51. These data suggest that HMGB1‐triggered signalling is sufficient to induce inter‐endothelial junction disruption. Finally, our data demonstrated that the inhibition of the activities of HMGB1 or caspase‐1 reversed visfatin‐induced changes in the membrane‐associated junction proteins. Thus, it is plausible that the instigation of endothelial Nlrp3 inflammasome by visfatin and its subsequent caspase‐1 activation triggers the release of HMGB1, which acts in an autocrine or paracrine fashion to promote inter‐endothelial junction disruption.

Previous studies have demonstrated HMGB1 can initiate cellular responses primarily by interacting with cell surface receptors RAGE and TLR4 leading to inflammation, immunity, chemotaxis and other cell processes 52, 53, 54, 55. In the present study, we found that down‐regulation of RAGE by gene silencing significantly attenuated visfatin‐induced disruption in tight and adherens junction in MVECs, whereas TLR4 siRNA had no effect. Our data suggest that visfatin‐induced inter‐endothelial junction disruption is mainly associated with HMGB1‐RAGE signalling in the endothelial cells. Our findings are consistent with recent studies showing that HMGB1 increased endothelial monolayer permeability or barrier dysfunction *via* RAGE signalling and multiple effector pathways 40, 56. In human umbilical vein endothelial cells, HMGB1 increases the endocytosis of membrane‐associated VE‐Cadherin and permeability of the endothelial cell monolayer *via* RAGE and Src Family Tyrosine Kinase pathways 40. In human lung endothelial cells, HMGB1 induced cell cytoskeletal rearrangement and barrier disruption *via* RAGE signalling, resulting in the downstream activation of p38 MAP kinase and phosphorylation of the actin‐binding protein Hsp27 56. Similar mechanisms may apply to the inter‐endothelial junction disruption by RAGE in our cell system. It should be noted that TLR4 has been reported to mediate HMGB1‐induced tissue factor expression in endothelial cells 57, 58. Thus, it seems that the interaction of HMGB1 with RAGE or TLR4 can cause different signalling cascades that may lead to distinct functional alterations in vascular cells.

We also perform animal experiments *in vivo* to determine whether HFD‐induced alteration in inter‐endothelial junction integrity is associated with endothelial inflammasome activation in the coronary arterial wall. Our findings demonstrate that treating mice with HFD decreased expression of tight junction and adherens junction protein, ZO‐1/2, occludin and VE‐Cadherin in endothelium layer of coronary arteries, whereas such HFD‐induced down‐regulation was prevented in mice with Nlrp3 gene deletion (Fig. 5). We further examined the inflammasome activation in coronary arterial endothelium by determining the relative activity of caspase‐1 using FLICA, a fluorescence probe which binds to active form of caspase‐1. To our knowledge, FLICA staining is the only method which directly measures caspase‐1 activity *in vivo*. Our data showed that HFD‐induced increases in caspase1 activity were mostly abolished in coronary arterial endothelium in mice when the Nlrp3 gene is deleted (Fig. 6). Our finding suggests that Nlrp3 inflammasome is the primary isoform of inflammasome that is activated in the coronary arterial endothelium by the HFD treatment. It should be noted that the staining of FLICA was not completely abolished by Nlrp3 gene deletion suggesting that other isoforms of inflammasome such as Nlrp1 or Nlrc4 may be involved in HFD‐induced inflammasome activation 59. Furthermore, mice received HFD showed enhanced HMGB1 expression in the intima of coronary arteries, which was abolished by Nlrp3 gene deletion (Fig. 7). Lastly, HFD‐induced T cell transmigration was abolished in coronary arteries when Nlrp3 gene is deleted supporting the view that down‐regulation of inter‐endothelial junction proteins causes junction disruption and increases endothelial permeability, and thereby facilitates T cell infiltration through endothelium (Fig. 8). Together, these data support the view that *in vivo* activation of endothelial Nlrp3 inflammasome by HFD can result in the release of HMGB1, which promotes the inter‐endothelial junction disruption in coronary arteries contributing to T cell adhesion and infiltration in the vasculature.

Instigation of the innate immune response by Nlrp3 inflammasomes is critical for the sterile inflammatory reaction to DAMPs during chronic degenerative diseases including metabolic diseases 60, 61. However, recent studies suggest that the inflammasomes activation may not only contribute to the inflammatory progression of these diseases. It has been shown that activation of inflammasomes directly produces a variety of the regulatory or pathogenic actions in cells or tissues beyond classical inflammatory response. These non‐classical actions of inflammasomes beyond inflammation include pyroptosis 62, 63, 64, interference with cellular cytoskeleton arrangement 65, alteration in cell membrane permeability 66 and enhanced lipid metabolism 50. Here, we demonstrate a novel non‐classical action that endothelial Nlrp3 inflammasome activation promotes disruption of inter‐endothelial tight and adherens junction integrity in metabolic diseases. Our findings add to the increasing evidence to support the view that non‐classical actions of inflammasomes, concurrent with the initiation of the innate immune response, may also be important in the regulation of cell function and in the mediation of different diseases 67.

In summary, we demonstrated that *in vitro*, activation of Nlrp3 inflammasome by injurious adipokine visfatin caused HMGB1 release and disruption of inter‐endothelial junctions in cultured MVECs. In a mouse model for diet‐induced obesity, HFD caused disruption of tight and adherens junction proteins in coronary arterial endothelium, which was accompanied by the activation of endothelial Nlrp3 inflammasome, enhanced expression of HMGB1 and T cell adhesion and infiltration in the coronary arterial walls. Our results, for the first time, demonstrate a novel direct action of endothelial Nlrp3 inflammasome on the development of endothelial dysfunction and injury during the early stage of metabolic disorders such as obesity or high‐fat/cholesterol diet.

## Conflicts of interest

None.

## Supporting information


**Figure S1** MVECs were transfected with scramble (Scr), TLR4 siRNA, or RAGE siRNA and incubated for another 24 hrs.
**Figure S2** HMGB1‐induced endothelial junction disruption effect is mediated by the RAGE.
