# Supplementary material for: Instigation of endothelial Nlrp3 inflammasome by adipokine visfatin promotes inter‐endothelial junction disruption: role of HMGB1
Source: J Cell Mol Med. 2015 Aug 20;19(12):2715–27. doi: 10.1111/jcmm.12657 (PMC4687695; doi:10.1111/jcmm.12657)
Supplement: Supplementary file 1 — Figure S1 MVECs were transfected with scramble (Scr), TLR4 siRNA, or RAGE siRNA and incubated for another 24 hrs. Figure S2 HMGB1‐induced endothelial junction disruption effect is mediated by the RAGE. [file JCMM-19-2715-s001.pdf]

Supplementary Material

Instigation of Endothelial Nlrp3 Inflammasome by Adipokine Visfatin Promotes Inter-endothelial Junction Disruption via HMGB1 Release

Yang Chen[1], Ashley L. Pitzer[1], Xiang Li[1,2], Pin-Lan Li[1], Lei Wang[1], Yang Zhang[1,2]

[1]Department of Pharmacology & Toxicology, School of Medicine, Virginia Commonwealth University,

Richmond, VA 23298

[2]Department of Pharmacological & Pharmaceutical Sciences, College of Pharmacy, University of Houston, Houston, TX 77204

Running title: Inflammasome-dependent endothelium junction disruption

Correspondence should be addressed to:

Yang Zhang, PhD., Department of Pharmacological & Pharmaceutical Sciences, College of Pharmacy, University of Houston, Houston, TX 77204-5056, Tel: 7137437710, Fax: 7137431259, Email: yzhan219@central.uh.edu

Supplementary Figure S1

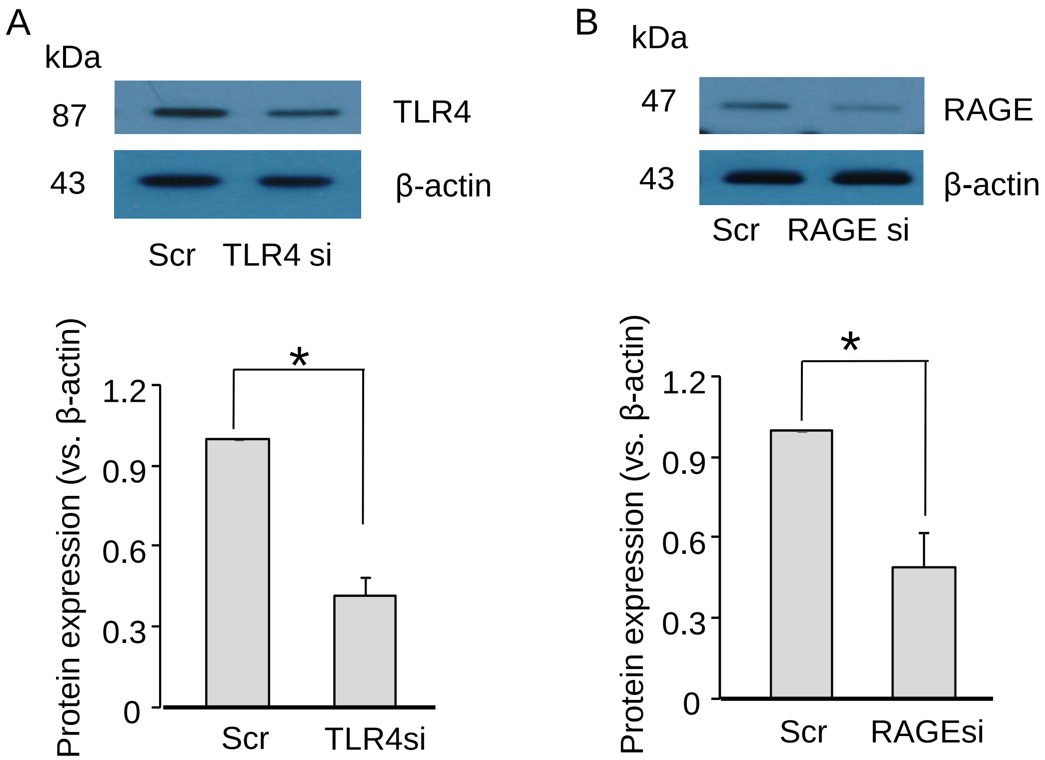

Figure S1. MVECs were transfected with scramble (Scr), TLR4 siRNA, or RAGE siRNA and incubated for another 24 hours. Representative Western blot gel documents and summarized data showing the effect of TLR4 (panel A) or RAGE (panel B) siRNA transfection on protein expression of TLR4 or RAGE genes in EOMA cells (n = 5). * $P<0.05$ *vs.* Scr.

Supplementary Figure S2

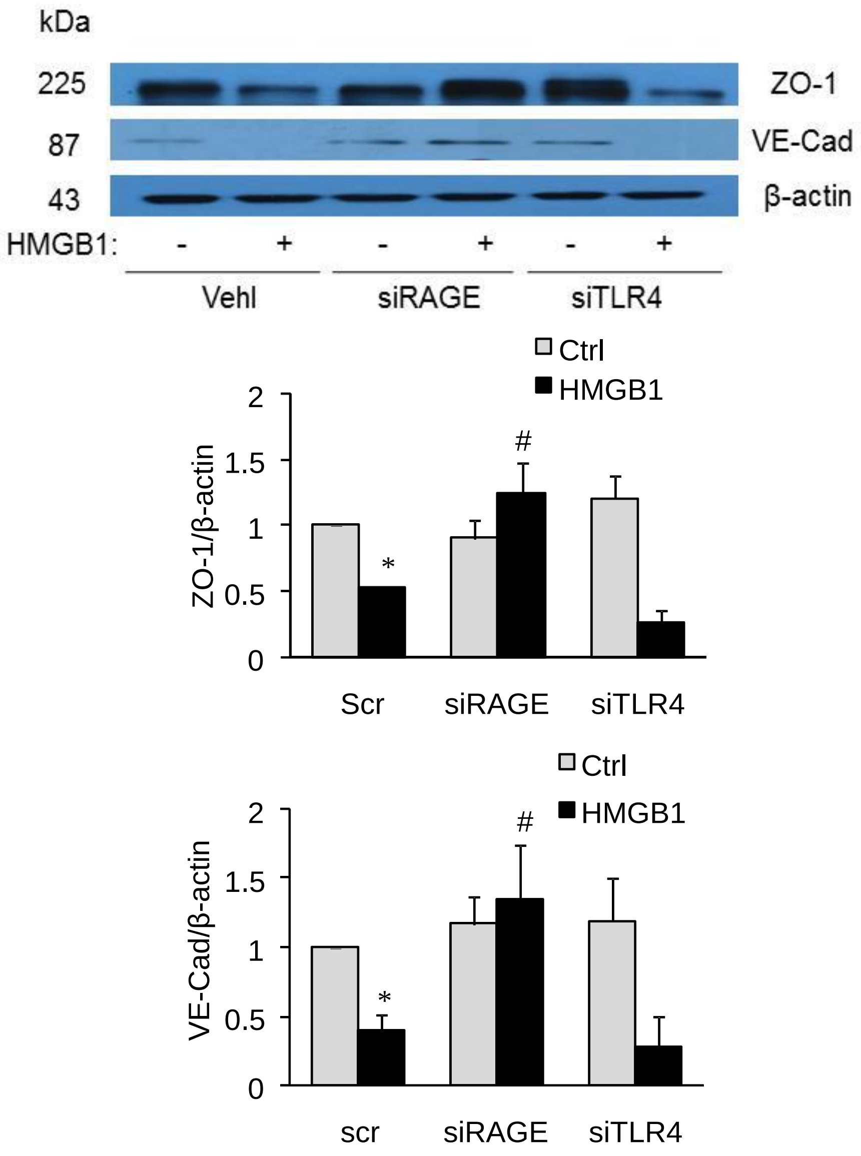

Figure S2. HMGB1-induced endothelial junction disruption effect is mediated by the RAGE. MVECs were transfected with scramble (Scr), RAGE siRNA (siRAGE) or TLR4 siRNA (siTLR4) for 4 hours and then stimulated with or without HMGB1 (15 µg/mL, 24 hours). Representative western blot gel document and summarized data showing the protein expression of ZO-1 and VE-Cadherin and β-actin expression in the microsomes of MVECs(n=3). * $P < 0.05$ vs. Scr Ctrl; # $P < 0.05$ vs. Scr+HMGB1.
